# The hypoxic microenvironment upgrades stem-like properties of ovarian cancer cells

**DOI:** 10.1186/1471-2407-12-201

**Published:** 2012-05-29

**Authors:** Dongming Liang, Yuanyuan Ma, Jian Liu, Claes Goran Trope, Ruth Holm, Jahn M Nesland, Zhenhe Suo

**Affiliations:** 1 Departments of Pathology, the Norwegian Radium Hospital, Oslo University Hospital, University of Oslo, Montebello, Ullernchausseen 70, N-0310, Oslo, Norway; 2 Departments of Gynaescology, the Norwegian Radium Hospital, Oslo University Hospital, University of Oslo, Montebello, Ullernchausseen 70, N-0310, Oslo, Norway; 3 Departments of Pathology, Institute for Clinical Medicine, Faculty of Medicine, University of Oslo, Montebello, Ullernchausseen 70, N-0310, Oslo, Norway; 4 Departments of Gynaecology, Institute for Clinical Medicine, Faculty of Medicine, University of Oslo, Montebello, Ullernchausseen 70, N-0310, Oslo, Norway; 5 Department of Pathology, Basic Medical College, The First Teaching Hospital of Zhengzhou University, Zhengzhou University, Zhengzhou, Henan Province, China

## Abstract

**Background:**

To study whether hypoxia influences the stem-like properties of ovarian cancer cells and their biological behavior under hypoxia.

**Method:**

Ovarian cancer cell lines ES-2 and OVCAR-3 were cultivated in different oxygen tensions for proliferation, cell cycling and invasion analyses. The clonogenic potential of cells was examined by colony formation and sphere formation assays. Stem cell surface markers, SP and CD44^bright^ and CD44^dim^ cells were analyzed by flow cytometry. Protein expression of HIF-1α, HIF-2α, Ot3/4 and Sox2 were investigated by Western blotting.

**Results:**

Both cell lines cultivated at hypoxic condition grew relatively slowly with extended G0/G1 phase. However, if the cells were pre-treated under 1% O_2_ for 48 hrs before brought back to normoxia, the cells showed significantly higher proliferation rate with higher infiltration capability, and significant more colonies and spheres, in comparison to the cells always cultivated under normoxia. CD44^bright^ cells expressed significantly higher levels of Oct3/4 and Sox2 than the CD44^dim^ cells and formed significantly more clones and spheres examined *in vitro*. Hypoxic treatment of the cells resulted in stronger CD44 expression in both cell lines, and stronger CD133 expression in the OVCAR-3 cell line. In parallel with these findings, significantly increased number of side population (SP) cells and up-regulated expression of Oct3/4 and Sox2 in both ES-2 and OVCAR-3 cell lines were observed.

**Conclusion:**

We conclude that ovarian cancer cells survive hypoxia by upgrading their stem-like properties through up-regulation of stemness-related factors and behave more aggressively when brought back to higher oxygen environment.

## Background

Hypoxic microenvironments are frequently found in many solid tumors including breast cancer, prostate cancer, brain tumor, malignant melanomas, metastatic liver cancer and ovarian cancer [1-5]. Solid tumors frequently encounter hypoxic stress. Rapidly proliferating cancer cells may outgrow their vascular network and limiting O_2_ diffusion within the tumors. Hypoxic stress can also be caused by perfusion defects as a result of abnormal tumor blood vessel structure and function [6]. Hypoxia not only accounts for tissue necrosis but also has a strong impact on tumor cell biology, with a decreased sensitivity to apoptotic and other cell-death signals, and increased signaling to promote angiogenesis, proliferation and systemic metastasis capacity [7]. Tumor hypoxia is not only a major problem for radiation therapy, but it has also been implicated in the development of resistance to many conventional chemotherapeutic agents [8,9]. The cancer stem cells particularly have been demonstrated to escape from the radiotherapy and chemotherapy and are able to form metastatic tumor in other organs. Several somatic tumors including ovarian tumors are considered to contain a small subset of stem-like cells called cancer stem cells, which have the capacity to self-renewal, differentiate and initiate new tumor [10-17].

The hypoxia inducible factors (HIFs) can be regulated by oxygen availability. HIFs are recognized as key modulators of the transcriptional response to hypoxic stress. Besides its adaptive function in cellular stress responses, recent work has also revealed important roles for HIFs in both physiological and pathological processes [6]. Increasing evidence indicates that HIFs regulate a number of genes including glucose metabolism, cell survival, erythropoiesis, stem cell maintenance, angiogenesis related markers and resistance to chemotherapy and radiation therapy [18]. Embryonic stem cell markers, the transcription factors Oct3/4 (also called POU5F1) and Sox2 have a pivotal role in the maintenance of self-renewal of embryonic stem cells and primordial germ cells. Oct3/4 is a homeodomain transcription factor of the POU family. Some laboratories found that the expression of Oct3/4 and Sox2 has an important role in cancer cells survival, self-renew, differentiation and proliferation in different somatic tumors such as lung, gastric, colorectal, rectal, bladder, breast, prostate and ovarian cancers [19-21].

In addition, cancer stem cells typically represent a small number of the total tumor cells which can be enriched on the basis of cell surface maker expression. Several surface markers have been reported to be associated with cancer stem-like or progenitor cells. In the ovarian cancer, the early progenitor cells are associated with some specific surface markers like CD44, CD133 and CD117 [22-26]. However, CD44^+^ and CD133^+^ subpopulations in ovarian cancer was believed to be heterogeneous and consisted of progenitor cells and differentiated cells as well [24,27].

Previously, SP cells have been found with some properties of cancer stem cells [28,29] and cancer stem cells also express ATP-binding cassette glycoprotein transporters on their surface. These transporters effectively pump out vital dyes, resulting in a characteristic unlabeled side population of cells detected in fluorescence activated cell sorting FACS plot.

The effect of hypoxia on the cancer stem-like characteristics of ovarian cancer cells has not been fully elucidated. Therefore, the aim of this study was to examine whether hypoxia can influence stem-like properties of ovarian cancer cell lines *in vitro* with the methods of proliferation assay, cell cycle analysis, infiltration assay, colony formation assay, sphere formation assay, SP, FACS and Western blotting. It was repeatedly shown that ovarian cancer cell lines OVCAR-3 and ES-2 under hypoxia exhibit significantly higher levels of stem-like features *in vitro.*

## Materials and methods

### Cell lines and cell cultures

Human ovarian cancer cell lines ES-2 and OVCAR-3 were purchased from American Type Culture Collection (ATCC, Manassas, VA, USA) and maintained in our lab for this study. For conventional cell culture, 2 × 10^5^ cells were seeded in 25 cm^2^ culture flasks and maintained in RPMI 1640 medium (Invitrogen, Carlsbad, CA, USA) supplemented with 10% fetal bovine serum (Invitrogen) and 100units/ml penicillin and 100 μg/ml streptomycin in a humidified 5% CO_2_ incubators at 37 °C.

### Hypoxic cell cultures

The Xvivo Closed Incubation System (XVIVO system 300 C, Biospherix, USA) was used in this study to obtain accurate oxygen tensions in different incubators. After 24 hrs cultivation in conventional cell culture (allowing cells to attach onto the flasks), the cells were transferred into different chambers with different oxygen controls for variable period of culture before the cells were harvested for additional examinations including MTT, sphere and colony formation assays, cell cycle analyses, flow cytometry analyses, Western blotting, SP and FACS.

### MTT assay

For evaluation of hypoxia influence on proliferation of cells, 2000 cells/well in 180 μl of complete RPMI-1640 medium were seeded into 96-well microplates under either normoxic (20% O_2_) or hypoxic (1% O_2_) conditions for variable time periods of culture before MTT analyses. In addition, to study whether hypoxia pretreatment influences the proliferation of cells differently, the cells were firstly pretreated under normoxic (20% O_2_) or hypoxic (1% O_2_) condition for 48 hrs before the cells were re-harvested and then cultured under normoxia for periods of times before MTT assay. After the cells in culture reached their time schedule, 5 mg/ml of 3-(4, 5-dimethylthiazol-2-yl)-2, 5-diphenyltetrazolium bromides (MTT, Sigma-Aldrich, St. Louis, MO;USA) was added and incubated at 37 °C for 4 hrs before 150 μL of dimethyl sulfoxide (DMSO) (Sigma-Aldrich) was added to each well and mixed thoroughly. The plates were then shaken for 15 min and absorbance was determined using a spectrophotometer at a wavelength of 490 nm (μQuant; Bio-Tek Instruments, Winooski, VT,USA).

### Cell cycle analysis

Cells were cultivated under hypoxia or normoxia for 48 hrs, and then harvested into 15 ml sterile conicaltubes, centrifuged and washed with ice-cold PBS. After cell counting, 1 × 10^6^ cells were fixed in 70% ethanol for 24 hrs at −20 °C, washed with cold PBS and re-suspended in PBS buffer containing 50 μg/ml of PI and 100 units/ml of RNase type A. The cells were then incubated in dark for 30 min at room temperature. Samples were filtered using a 70 μm nylon membrane and analyzed with an LSRII flow cytometer (Becton Dickinson, San Jose, CA, USA) after gating was optimized. Data were analyzed with the FlowJo software (Version 7.6).

### Invasion assay

The invasion assay was performed with 24-transwell chambers (Costar, Bodenheim, Germany), as previously described [30]. Briefly, 1x10^4^ cells were harvested and re-suspended in 200 μl RPMI-1640 medium without serum before plated in the top chamber. The lower chamber of the transwell was filled with 500 μl RPMI-1640 medium supplemented with 10% fetal bovine serum. The cell suspension was applied onto the matrigel membrane and incubated at 37 °C for 24 hrs. The cells migrated through the matrigel and the filter were fixed with 70% methanol, stained with 0.2% crystal violet, washed with ddH_2_O and counted under microscopy.

### Sphere formation assay

Sphere formation assay was performed based on the previously described method [31]. The ES-2 and OVCAR-3 cells were plated at different oxygen tensions for 48 hrs, and then dispatched from cell culture flask and harvested. Single cells (1000 cells per well) were re-plated and cultivated under normoxia at ultralow attachment six-well plates (ultra low cluster plates, Life sciences). These cells were cultivated for 14 days under normoxic condition before the spheres were evaluated under inverse miscopy and counted (more than 30 cells within a sphere was considered to be a full sphere). In addition, sphere formation assays of single CD44^bright^ and CD44^dim^ cells (800 cells per well) were also performed with the method as described above. All the experiments were repeated three times.

### Colony formation assay

The cells in 80% confluent were cultivated in different oxygen conditions for 48 hrs and then washed with PBS and dispatched from cell culture flask, harvested and counted with the Countess Cell Counter (Invitrogen). 500 cells/well were plated in 6-well plates and placed at 20% oxygen tension for 14 days, and the colonies were fixed with 4% buffered formalin for 15 min and then stained with 1% crystal violet for 30 min. The plates were gently washed with PBS and dried before colony evaluation under microscopy. In addition, the CD44^bright^ and CD44^dim^ cells (500 cells/well) were also performed by colony formation assay with the method as described above. Colonies which contained more than 30 cells was counted. Colony formation efficiency was calculated as following: colony numbers/seed cells number × 100%. Data are representative of three independent experiments.

### Flow cytometry and FACS

After 48 hrs incubation under hypoxia (1% O_2_) or normoxia (20% O_2_), the cells were trypsinized, counted and washed with cold FACS buffer (PBS + BSA 0.02%) with final concentration of 1 × 10^6^ cells/tube. The cells were pre-blocked with 0.5% BSA for 30 min on ice before incubation for 30 min in the dark, on ice with CD44 monoclonal antibody directly conjugated with allophycoyanin (APC) and CD133 monoclonal antibody directly conjugated with fluorescein isothiocyanate (FITC) both obtained from BD Pharmingen Company. Following washing twice with ice-cold PBS, the cell suspensions in 800 μl FACS buffer were filtered through a 70 mm nylon mesh. Samples were analyzed on a LSRII flow cytometer (Becton Dickinson, San Jose, CA, USA). Viable and single cells were gated for each sample acquired and APC Mouse IgG2b and FITC Mouse IgG2b (both from BD Pharmingen, USA) were used as negative control. The experiments were performed at three independent times. Data were analyzed using FlowJo software (Version 7.6).

For FACS, the cells were dissociated to viable single cell suspensions and stained with CD44 monoclonal antibody directly conjugated with APC as described above. Corresponding non-immune isotype was included as negative control. Top 10% CD44^+^ were separated with FACS and determined as CD44^bright^ cells and bottom 10% of CD44^-^ cells were separated with FACS and determined as CD44^dim^ cells. FACS was performed with a FACSAria flow cytometer (Becton Dickinson).

### SP analysis

For SP analysis, 1 × 10^6^ cells were suspended in pre-warmed RPMI 1640 medium containing 2% fetal bovine serum and 2 mM HEPES buffer. Hoechst 33342 dye (Sigma-Aldrich) was added to a final concentration of 5 μg/ml from a stock solution of 1 mg/ml in presence or absence of verapamil (50 μM; Sigma) and incubated at 37 °C for 90 min with intermittent shaking. At the end of the incubation, the cells were washed with ice-cold HBSS with 2% FBS, centrifuged at 4 °C and re-suspended in ice-cold HBSS containing 2% FBS. Propidium iodide at a final concentration of 2 μg/ml was added to the cells to gate viable cells. The cells were filtered through a 70-μm cell strainer to obtain single cell suspension before analyzed with a LSRII flow cytometer (BD Biosciences, Franklin Lakes, NJ, USA). Cell aggregates were discarded from the analysis by doublet discrimination. SP cells were visualized or sorted by use of red (blue, 402–446 nm) *vs* blue (red, 650–670) ultraviolet channels both in linear mode.

### Western blot analysis

Cultured cells were harvested and lysed with 200 μl of whole cell protein RIPA buffer (25 mM Tris HCl pH 7.6, 100 mM NaCl, 1% NP40, 1% Sodium deoxycholate, 0.1% SDS, Thermo Scientific Pierce, Bonn, Germany) with added freshly prepared proteinase inhibitors (0.1 μM Aprotinin, 1.0 mM PMSF, 1 μM Leupeptin, 1 μM Pepstatin), and then the samples were frozen at −70 °C for 30 min. The samples were centrifuged at 15,000 *rpm* for 15 min at 4 °C and the supernatants were transferred to new tubes. The protein concentrations were measured with Bio-Rad protein assay (Hercules, CA, USA) according to the manufacturer’s instruction. After heated with a benchtop heater (Model 111002, Boekel Scientific, Feasterville, PA, USA) at 100 °C for 5 min in SDS-loading buffer (500 mM Tris HCl pH 6.8; 10% Glycerol, 2% SDS, 0.6 M DTT, 0.05% Bromphenol blue), 50 μg protein per sample was subjected to 10% SDS-PAGE and transferred to polyvinylidene difluoride transfer membrane (Bio-Rad). Membranes were blocked with 5% non-fat dry milk in 0.05% TBS-Tween for 90 min at room temperature and incubated overnight at 4 °C with the primary antibodies against GAPDH (0.2 μg/ml), Oct3/4 (1 μg/ml), Sox2 (1 μg/ml), HIF-1α (1 μg/ml) and HIF-2α (1 μg/ml) all from R&D Systems, Minneapolis, MN, USA. The membranes were then incubated with corresponding secondary HRP-conjugated antibodies including anti-goat IgG-HRP antibody (1:2000) or anti-mouse IgG-HRP antibody (1:1000) all from R&D Systems, Minneapolis, MN, USA. Immuno-complexes were visualized by enhanced chemiluminescence (GE Healthcare, Bucks, and UK). The western blotting experiments were repeated at least three times.

### Statistical analyses

Data are shown as mean ± SEM of at least 3 experiments for each experiment. SPSS software (version 16.0) was used for data analysis. Statistical analysis was performed using Student’s *T* test (*P* < 0.05 was considered statistically significant.

## Results

### Hypoxia results in comparatively slower growth rate with extended G0/G1 cell cycle

**Figure 1:**
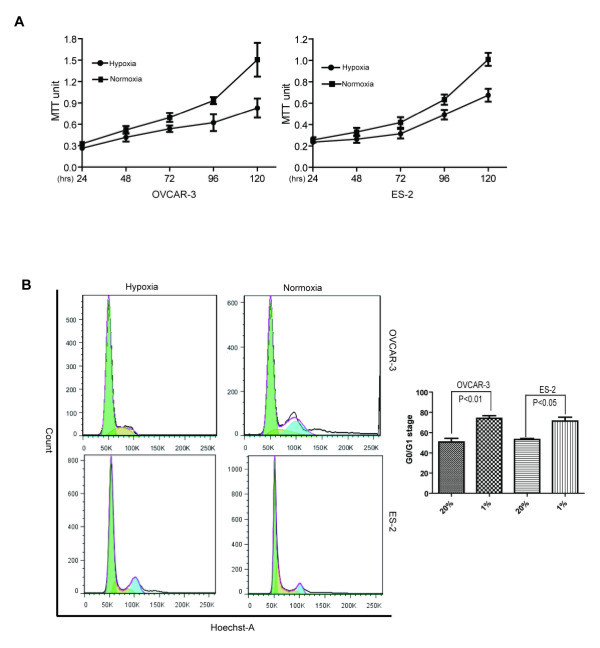
**Hypoxia extended G0/G1 status with relatively slow growth of cells.** Both OVCAR-3 and ES-2 cells were maintained under either hypoxia or normoxia for variable periods of times. **(A)** Growth curves show that the cells under hypoxia grew relatively slower than those cells under normoxia (*P* < 0.01). **(B)** Cell cycle analyses show extended G0/G1 phase in the cells cultivated under hypoxia, and the corresponding histogram shows statistically significantly increased G0/G1 stage in both cell lines (*P* < 0.01 for OVCAR-3 and *P* < 0.05 for ES-2).

ES-2 and OVCAR-3 ovarian cancer cell lines were cultivated under either hypoxia or normoxia for periods of times by MTT assay to investigate the effect of hypoxia on cell growth. As shown in Figure 1A, the cells cultivated under 1% O_2_ (hypoxia) grew relatively slowly than the cells cultivated under normoxia (*P* < 0.01), although the growth difference was not so apparent in the first 48 hrs, indicating that the cell growth was inhibited under hypoxic condition. The cells cultivated under either hypoxia or normoxia were then examined with cell cycle analysis. It could be repeatedly shown that there was significantly increasing number of G0/G1 phase cells in the OVCAR-3 and ES-2 cells, from 50.8 ± 6.2% and 53.17 ± 1.98% in the cells cultivated under normoxia compared to 74.1 ± 4.5% and 71.44 ± 6.6% in the cells cultivated under hypoxia, respectively (Figure 1B). As shown in the histogram, there was statistically significant difference for G0/G1 stage in both cell lines (*P* < 0.01 for OVCAR-3 and *P* < 0.05 for ES-2), indicating more quiescent cells under hypoxia condition.

### Hypoxic pretreatment promotes cell proliferation and invasion

**Figure 2:**
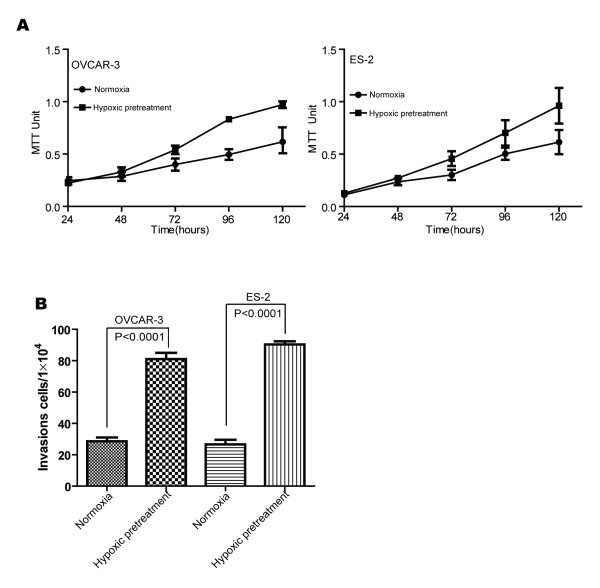
**Hypoxia pretreatment induced cell proliferation and invasion potential.** All the cells were cultivated under either hypoxia or normoxia for 48 hrs before brought back to normoxia condition for proliferation or invasion assays. **(A)** The cells with hypoxia pretreatment for 48 hrs show significantly faster in growth than the cells always cultivated under normoxia (*P* < 0.05). **(B)** Cell invasion assays show that there are significantly more infiltrated cells in the hypoxia pretreated groups than those in the normoxia control groups in both cell lines (*P* < 0.0001).

To explore how hypoxia-pretreatment influenced the biological behaviors of the cells, proliferation and invasion assays were performed. As shown in Figure 2A, all the hypoxia pretreated cells grow faster than those control cells always cultivated in normoxia. When the cells were pretreated under hypoxia for 48 hrs and then cultivated under normoxia, MTT values of both cell lines were significantly higher than those cells always kept under normoxia (*P* < 0.05). Furthermore, our invasive assays revealed significantly higher number of invasive cells in the hypoxia pretreated cells, both in the OVCAR-3 and ES-2 cells. As shown in Figure 2B, there are 2.9-fold increase in number of invasive cells in the OVCAR-3 cells under hypoxia and 3.5-fold increase in number of invasive cells in the ES-2 cells under hypoxia (*P* < 0.0001 for both cell lines).

### Hypoxia increases sphere and colony formation capability of the cells

**Figure 3:**
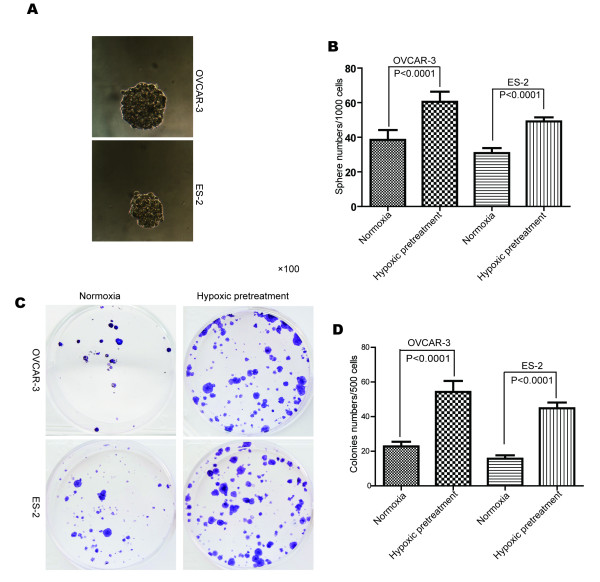
**Hypoxia pretreatment increased the sphere and colony formation capability of cells.(A)** Figure 3A shows representative spheres of the cells cultivated under normoxia. **(B)** Results from three independent experiments show significantly more spheres in the hypoxia pre-treated groups than the control groups for both cell lines (*P* < 0.0001). **(C)** Colony formation assay shows more colonies in the hypoxia pre-treated cells. **(D)** Results from three independent experiments show significantly more colonies in the hypoxia pre-treated groups than those in the normoxia controls (*P* < 0.0001).

Under normoxia condition, spheres could be observed in both cell lines (Figure 3A). Comparatively, hypoxia pretreatment increased the number of spheres, with 1.96-fold increase in the VCAR-3 cells and 1.59-fold increase in the ES-2 cells (Figure 3B). The cells with hypoxia pretreatment were also examined with the colony formation assay. Compared with the cells always kept in normoxia, more colonies were observed in the hypoxia pretreated cells (Figure 3C), with 2.38-fold increase in the OVCAR-3 cells and 2.86-fold increase in the ES-2 cells, respectively (Figure 3D).

### Hypoxia upregulates the expression of CD44 and CD133

**Figure 4:**
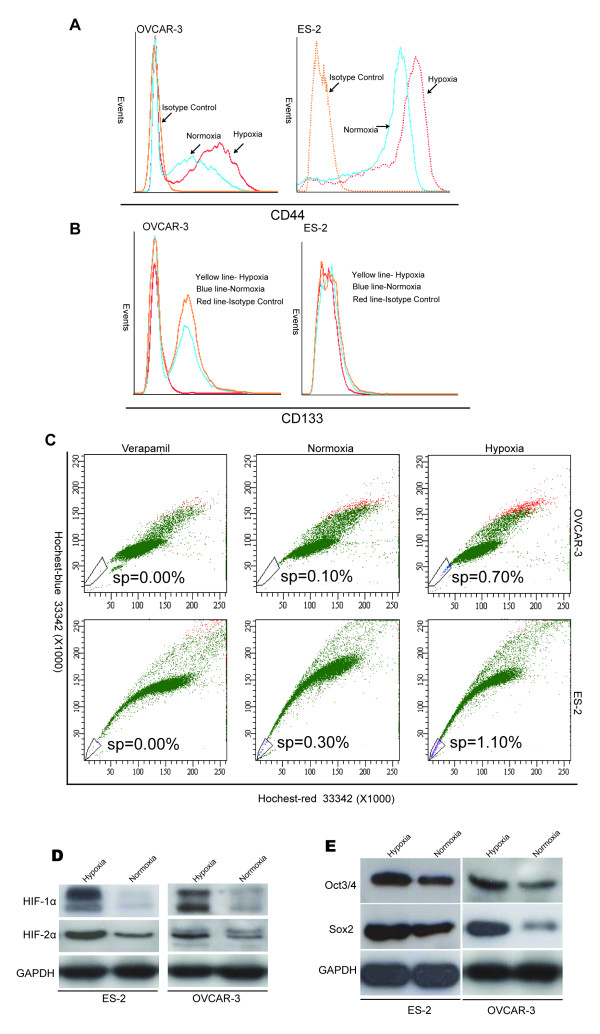
**Hypoxia induced molecular stem-like phenotype.** The ovarian cancer cells were maintained under either hypoxia (1% O_2_) or normoxia for 48 hrs before additional analyses. **(A)** Comparatively, there were significantly higher levels of CD44 expression in the OVCAR-3 and ES-2 cells cultivated under hypoxia. **(B)** There were also significantly higher levels of CD133 expression in the hypoxia cultivated OVCAR-3 cells, but the increase of CD133 expression in ES-2 cells was not apparent. **(C)** SP assay shows also significantly higher number of SP cells in both the OVCAR-3 and ES-2 cells under hypoxia. **(D)** The HIF-1α and HIF2-α expressions were induced in the hypoxia cultivated OVCAR-3 and ES-2 cells; **(E)** The expressions of Oct3/4 and Sox2 were also correspondingly increased in the hypoxia cultivated OVCAR-3 and ES-2 cells.

The surface markers CD44 and CD133 were examined by flow cytometry in consideration of the effect of hypoxia. There were 1.99-fold increase in CD44 expression in the OVCAR-3 cells under hypoxia and 2.73 fold increase in CD44 expression in the hypoxia cultivated ES-2 cells (Figure 4A). CD133 expression in the OVCAR-3 cells was also increased with 1.48-fold, while almost no change was observed in the ES-2 cells under hypoxia (Figure 4B). All these data indicate that hypoxia induces the expression of CD44 expression in these cell lines, and also positively influences the expression of CD133, at least in the OVCAR-3 cells.

### Hypoxia enriches SP cells

To determine whether hypoxia affected stemness properties in ovarian cancer cells, the SP assay was performed in the ovarian cancer cell lines OVCAR-3 and ES-2 cells cultivated either under normoxia (20% O_2_) or under hypoxia (1% O_2_) for 48 hrs. As shown in Figure 4C hypoxia could enrich SP cells in both the OVCAR-3 and ES-2 cells, with about 7-fold increase in the hypoxia cultivated OVCAR-3 cells and 3.7-fold increase in the hypoxia cultivated ES-2 cells, compared to the cells cultivated under normxia.

### Hypoxia up-regulates HIFs and transcription factors

Hypoxia-inducible factors are a major family of transcriptional factors activated by hypoxia. The effect of hypoxia on the HIF-1α and HIF-2α expressions was investigated in the ovarian cancer cell lines. The expression of HIF-1α and HIF-2α in both the OVCAR-3 and ES-2 cells were significantly increased in the cells cultivated under hypoxia for 48 hrs, whereas the cells cultivated under normoxia for 48 hrs were only weakly positive (Figure 4D). In the normoxia cultures the level of HIF-2α was higher than the level of HIF-1α. The expression of both Oct3/4 and Sox2 was significantly increased in the ES-2 and OVCAR-3 cells cultivated in the hypoxia condition for 48 hrs, in comparison to the cells cultivated in normoxia condition (Figure 4E).

### CD44^bright^ cells display stem-like properties

**Figure 5:**
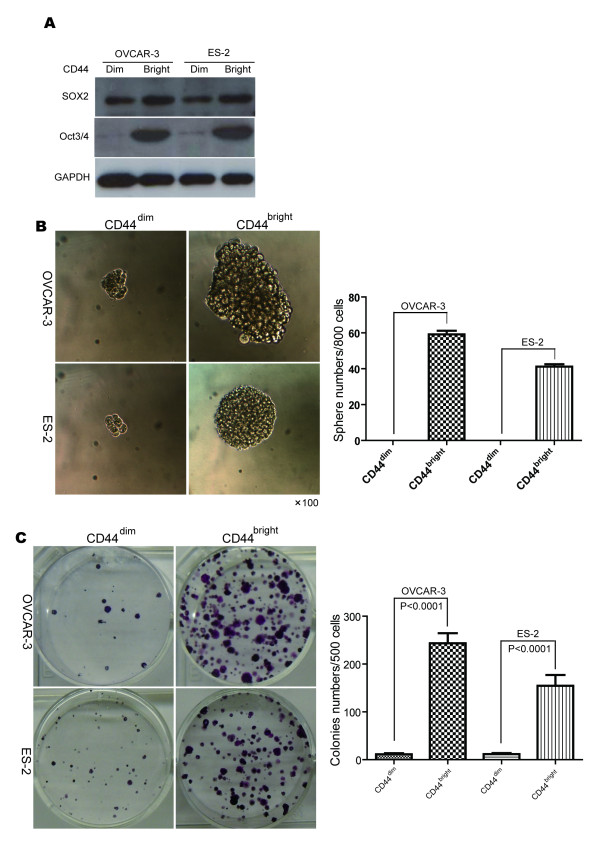
**CD44**^**bright**^**cells show stem-like properties.** The CD44^bright^ and CD44^dim^ cells were sorted by FACS from the OVCAR-3 and ES-2 cells before additional Western blotting, sphere and colony formation assays. **(A)** The CD44^bright^ cells expressed higher levels of Sox2 and Oct3/4 than the CD44^dim^ cells in both cell lines. **(B)** Larger spheres from the CD44^bright^ cells and cell clusters not qualified as spheres from the CD44^dim^ cells in both cell lines are shown (left panel) with a corresponding histogram (right panel). **(C)** Colony formation assay also shows more colonies in the CD44^bright^ cells compared to the CD44^dim^ cells in both cell lines (left panel). Three independent colony formation assays show statistically significantly more colonies in the CD44^bright^ cells, in comparison to the CD44^dim^ cells in both cell lines (*P* < 0.0001, right panel).

We discovered during our experiments that the SP cells separated by FACS were difficult to maintain *in vitro*, most probably due to the chemical damage of the Hoechst 33342 dye. In addition, the CD133 expression was not repeatedly elevated in the ES-2 cells under hypoxia. Therefore, we decided to further study the CD44^bright^ cells in special consideration of their stemness features *in vitro*. As shown in Figure 5A, the CD44^bright^ ovarian cancer cells expressed high levels of Oct3/4, whereas the CD44^dim^ cells expressed almost no Oct3/4 in both cell lines. The CD44^bright^ cells expressed higher levels of Sox2 than the CD44^dim^ cells.

In sphere formation assay, there was almost no qualified sphere in the CD44^dim^ cells, whereas more spheres were observed in the CD44^bright^ cells (Figure 5A and B). In addition, significantly more colonies could be observed in the CD44^bright^ cells than the CD44^dim^ cells examined with the method of colony formation assay (Figure 5C, *P* < 0.0001).

## Discussion

Hypoxia is an inherent characteristic of many solid tumors. It is well known pathologically that tumor cells tolerate hypoxic environment. It has been documented in embryonic and adult stem cell research that hypoxia favors cell growth *in vitro*[32-35]. However for cancer cells, it is not fully studied how hypoxia influences the stem-like properties, although there has been a great progress in this field [18,36-39]. Therefore we explored whether hypoxia is a driving force for the growth of ovarian cancer cells *in vitro* by performing MTT experiment. It was found that although the growth difference was not apparent during the first 48 hrs, the cells in the hypoxic condition grew generally slower that the corresponding cells under normoxia. The cells cultivated under hypoxia were further investigated by cell cycle analysis. We discovered that both ES-2 and OVCAR-3 cell lines experienced a significant G0/G1 phase extension under hypoxia for 48 hrs, indicating more quiescent cells under hypoxia. This result is in line with other tumor cell studies [40-42]. Theoretically, cancer stem cells should have a low rate of division and proliferation in their niche which may help to decrease their chemotherapy and radiotherapy sensitivity [3,8,38].

Then we focused on the effect of hypoxia pretreatment followed by normoxia cultivation in the ovarian tumor cell lines, since in present study we found that the cells grew poor if always placed under hypoxia. The tumor cells were placed under 1% O_2_ for 48 hrs as hypoxia pretreatment group before they were brought back to normoxia, with the cells always under normoxia as control. We found that the hypoxia pretreated tumor cells followed by normoxia cultivation grew significantly faster with significantly higher infiltration capability in comparison to the cells always in normoxia. These results indicate that cancer cells may switch into a more stem-like status when meeting with hypoxic stress, and develop more aggressive phenotype in a manner of selection in a suddenly higher oxygen environment, such as when tumor cells penetrate into blood stream or when the “dormant” metastatic solid tumor cells are mobilized out of bone marrow by till now un-clarified mechanisms. This may be useful to explain why hypoxia not only accounts for tissue necrosis, but also a strong impact on tumor cell biology, with a decreased sensitivity to apoptotic and other cell-death signals, and an increased signaling to promote angiogenesis, proliferation and systemic metastasis capacity when the niche permits [43-46].

It is known that cancer stem cells residing in epithelial ovarian cancers can be CD133^+^[24] or CD44^+^ subpopulation cells [26] and these specific markers may be potential therapeutic targets in this devastating disease. Therefore the influence of hypoxia on the expression of these two markers was explored in this study. Our results show that 48 hrs 1% O_2_ treatment could result in about 2.0-2.7-fold increase in CD44 expression in both ES-2 and OVCAR-3 cell lines, and about 1.5-fold increase in CD133 expression in the OVCAR-3 cell line. These results are supported by other reports that hypoxia helps to expand the CD133^+^ pancreatic cancer cells [47] and CD133^+^ glioma stem cells [48]. It is also reported that hypoxia enriches the CD44^+^/CD24^-^ breast cancer stem-like cells [49] and CD44^+^ murine mesenchymal stem cells [47-50].

To study the effect of hypoxia on stem-like cell phenotypes, we assessed the fraction of SP cells since SP assay has been successfully used for identification of cancer stem-like cells in hepatocellular, breast and ovarian cancers [14,23,29,51,52]. We found that hypoxia could induce the SP subpopulation in both OVCAR-3 cells and ES-2 cells. In addition, the increased SP cells were in parallel with increasing expression levels of HIF-1α and HIF-2α in both cell lines. It is known that hypoxia often occurs inside solid tumor and exhibits more severe at the undifferentiated parts of tumors compared to surrounding tumor or normal tissues. The initial response of cancer cells to hypoxia is the activation of hypoxia responsive transcription factor. The hypoxia inducible factors HIF-1α and HIF-2α are important factors activated under hypoxia. It has been reported that HIF-1α expression is increased in other ovarian cancer cell lines in response to hypoxia [42,53], which are largely in line with our present study. It has been observed that HIFs influence the phenotypes of tumors by regulating a number of target genes such as glucose transporters, glycolic enzyme, vascular endothelial cell growth and growth factors [16,18,54].

The stem-like property impact of hypoxia may be exerted through transcriptional factors, such as Oct3/4 [16,32] and Sox2 [55]. Oct3/4 and Sox2 are key players in a transcriptional network for maintenance of embryonic stem cell and primordial germ cells self-renewal. Several studies suggest a role for Oct3/4 and Sox2 in sustaining stem-like property of adult somatic stem cells [56,57]. Our study verifies that both Oct3/4 and Sox2 were weakly expressed in the OVCAR-3 and ES2 cell lines, and the expressions of these proteins were up-regulated upon hypoxia exposure. Furthermore, such increasing levels of Oct3/4 and Sox2 expression were in parallel with the increasing levels of HIF-1α and HIF-2α expression under hypoxia. Previous study show that HIF-2α binds to the promoter of Oct3/4 and directly induces its expression and activity [58], suggesting a potential role for the interaction of HIF-2α and Oct3/4 in ovarian cancer cells upon hypoxia environment.

Since CD44 has been indicated as a putative surface marker for cancer stem/progenitor cells in breast and ovarian cancers [23,26,27,49], and since its expression was significantly up-regulated in the OVCAR-3 and ES2 cell lines under hypoxia, we decided to further analyze whether CD44 expression was associated with any stem-like property in these cells. The expression of Oct3/4 in the CD44^dim^ cells was almost negative, while its expression in the corresponding CD44^bright^ cells was dramatically increased. We performed additional colony formation and sphere formation assays with the isolated corresponding CD44^dim^ and CD44^bright^ cells by FACS. Significantly more spheres and colonies could be repeatedly seen in the CD44^bright^ cells than those CD44^dim^ cells. Therefore, our results verify that hypoxia significantly increases the expression of CD44, and CD44^bright^ cells possess significantly higher stem-like properties in the ovarian cancer cell lines OVCAR-3 and ES2.

## Conclusion

In summary, our results reveal that ovarian cancer cells OVCAR-3 and ES2 under hypoxia showed extended G0/G1 phase, a more quiescent status, and more SP cells. At the same time these cells under hypoxia expressed higher levels of CD44, CD133, Oct3/4 and Sox2. It is further verified that CD44^bright^ cells contributed to the higher stem-like properties of the cells. If the cells were cultivated in 1% O_2_ for 48 hrs and then brought back to normoxia the cells demonstrated significantly higher growth rate with higher infiltration potential and significantly higher colony and sphere formation capability than those cells always under normoxia. It is concluded that ovarian cancer cells may survive hypoxia by upgrading their stem-like properties through up-regulation of stemness-related factors and behave more aggressively when brought back to higher oxygen environment.

## Competing interests

The authors declare that there are no competing interests.

## Authors’ contributions

DL carried out cell culture, MTT, colony formation and flow cytometry assays, analyzed the data and drafted the manuscript. YM performed cell culture, sphere formation and flow cytometry assays, analyzed the data and drafted the manuscript. JL performed the cell culture, western blotting experiments. CGT, RH and JMN participated in the experiment design, analyzed the data and modified the manuscript.

ZS contributed to the design of the experiment, analysed the data and gave final approbal of the version to be submitted.

All the authors have read and approved the final manuscript.

## Pre-publication history

The pre-publication history for this paper can be accessed here:

http://www.biomedcentral.com/1471-2407/12/201/prepub
